# 3-(4-Fluoro­phenyl­sulfin­yl)-2,4,6-trimethyl-1-benzofuran

**DOI:** 10.1107/S1600536810004836

**Published:** 2010-02-13

**Authors:** Hong Dae Choi, Pil Ja Seo, Byeng Wha Son, Uk Lee

**Affiliations:** a Department of Chemistry, Dongeui University, San 24 Kaya-dong Busanjin-gu, Busan 614-714, Republic of Korea; b Department of Chemistry, Pukyong National University, 599-1 Daeyeon 3-dong, Nam-gu, Busan 608-737, Republic of Korea

## Abstract

In the title compound, C_17_H_15_FO_2_S, the O atom and the 4-fluoro­phenyl group of the 4-fluoro­phenyl­sulfinyl substituent lie on opposite sides of the plane of the benzofuran; the 4-fluoro­phenyl ring is almost perpendicular to this plane, making a dihedral angle of 88.99 (4)°. The crystal structure exhibits inter­molecular C—H⋯O hydrogen bonds and C—H⋯π inter­actions between the methyl H atom and the 4-fluoro­phenyl ring.

## Related literature

For the crystal structures of similar 2-methyl-3-phenyl­sulfinyl-1-benzofuran derivatives, see: Choi *et al.* (2007 ▶, 2008**a* ▶,b* ▶). For the pharmacological activity of benzofuran compounds, see: Aslam *et al.* (2006 ▶); Galal *et al.* (2009 ▶); Khan *et al.* (2005 ▶). For natural products with benzofuran rings, see: Akgul & Anil (2003 ▶); Soekamto *et al.* (2003 ▶).
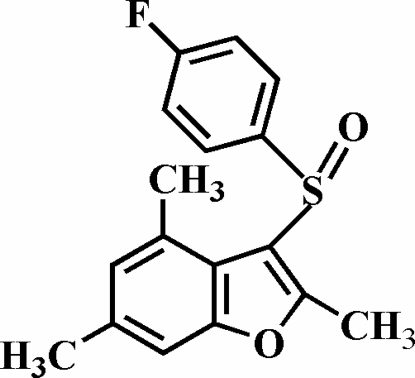


## Experimental

### 

#### Crystal data

C_17_H_15_FO_2_S
*M* *_r_* = 302.35
Orthorhombic, 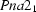

*a* = 11.9486 (4) Å
*b* = 18.8134 (9) Å
*c* = 6.4435 (3) Å
*V* = 1448.46 (11) Å^3^
*Z* = 4
Mo *K*α radiation
μ = 0.24 mm^−1^
*T* = 173 K
0.36 × 0.34 × 0.23 mm

#### Data collection

Bruker SMART APEXII CCD diffractometer
Absorption correction: multi-scan (*SADABS*; Bruker, 2009 ▶) *T*_min_ = 0.920, *T*_max_ = 0.947
6188 measured reflections
2147 independent reflections
2094 reflections with *I* > 2σ(*I*)
*R*_int_ = 0.027

#### Refinement

*R*[*F* ^2^ > 2σ(*F* ^2^)] = 0.029
*wR*(*F* ^2^) = 0.076
*S* = 1.05
2147 reflections
193 parameters
1 restraint
H-atom parameters constrained
Δρ_max_ = 0.19 e Å^−3^
Δρ_min_ = −0.32 e Å^−3^
Absolute structure: Flack (1983 ▶), 745 Friedel pairs
Flack parameter: 0.01 (8)

### 

Data collection: *APEX2* (Bruker, 2009 ▶); cell refinement: *SAINT* (Bruker, 2009 ▶); data reduction: *SAINT*; program(s) used to solve structure: *SHELXS97* (Sheldrick, 2008 ▶); program(s) used to refine structure: *SHELXL97* (Sheldrick, 2008 ▶); molecular graphics: *ORTEP-3* (Farrugia, 1997 ▶) and *DIAMOND* (Brandenburg, 1998 ▶); software used to prepare material for publication: *SHELXL97*.

## Supplementary Material

Crystal structure: contains datablocks I. DOI: 10.1107/S1600536810004836/fk2013sup1.cif

Structure factors: contains datablocks I. DOI: 10.1107/S1600536810004836/fk2013Isup2.hkl

Additional supplementary materials:  crystallographic information; 3D view; checkCIF report

## Figures and Tables

**Table 1:** Hydrogen-bond geometry (Å, °) *Cg* is the centroid of the C11–C16 ring.

*D*—H⋯*A*	*D*—H	H⋯*A*	*D*⋯*A*	*D*—H⋯*A*
C10—H10*A*⋯O2^i^	0.98	2.60	3.301 (3)	129
C10—H10*C*⋯*Cg*^ii^	0.98	2.78	3.604 (3)	142

Symmetry codes: (i) 
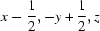
; (ii) 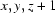.

**Fig. 1.**
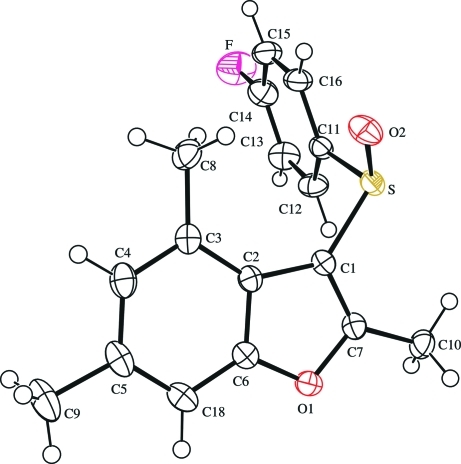
The molecular structure of the title compound with the atom numbering scheme. Displacement ellipsoids are drawn at the 50 % probability level. H atoms are presented as a small spheres of arbitrary radius.

**Fig. 2.**
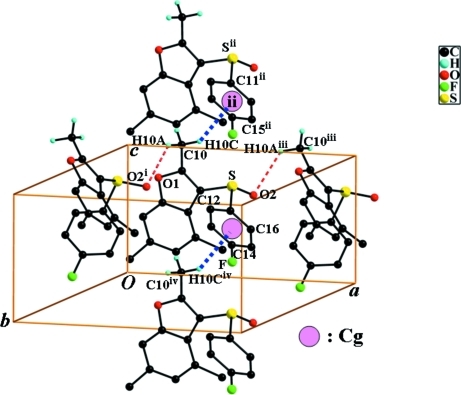
C—H···O and C—H···π interactions (dotted lines) in the crystal structure of the title compound. Cg denotes the ring centroid. [Symmetry codes: i) x - 1/2, - y + 1/2,z; ii) x, y, z + 1; iii) x + 1/2, - y + 1/2, z; iv) x, y, z - 1.]

## supplementary crystallographic information

### Comment

Molecules containing benzofuran skeleton show significant pharmacological activities such as antifungal (Aslam *et al.*,  2006), antitumor and antiviral (Galal *et al.*, 2009), antimicrobial (Khan *et al*., 2005) properties. These compounds are widely occurring in nature (Akgul & Anil, 2003; Soekamto *et al.*, 2003). As a part of our ongoing studies of the effect of side chain substituents on the solid state structures of 2-methyl-3-phenylsulfinyl-1-benzofuran analogues (Choi *et al.*, 2007, 2008*a,b*), we report the crystal structure of the title compound (Fig. 1).

The benzofuran unit is essentially planar, with a mean deviation of 0.013 (2) Å from the least-squares plane defined by the nine constituent atoms. The 4-fluorophenyl ring is almost perpendicular to the plane of the benzofuran fragment [88.99 (4)°] and is tilted slightly towards it. The crystal packing (Fig. 2) is stabilized by an intermolecular C—H···O hydrogen bond between the methyl H atom and the oxygen of the S═O unit, with a C10—H10A···O2^i^ (Table 1). The molecular packing (Fig. 2) is further stabilized by an intermolecular C—H···π interaction between the methyl H atom and the 4–fluorophenyl ring, with a C10—H10C···Cg^ii^ (Table 1; Cg is the centroid of the C11–C16 4-fluorophenyl ring).

### Experimental

77% 3-Chloroperoxybenzoic acid (224 mg, 1.0 mmol) was added in small portions to a stirred solution of 3-(4-fluorophenylsulfanyl)-2,4,6-trimethyl-1-benzofuran (257 mg, 0.9 mmol) in dichloromethane (30 mL) at 273 K. After being stirred at room temperature for 3h, the mixture was washed with saturated sodium bicarbonate solution and the organic layer was separated, dried over magnesium sulfate, filtered and concentrated in vacuum. The residue was purified by column chromatography (hexane-ethyl acetate, 1:1 v/v) to afford the title compound as a colorless solid [yield 82%, m.p. 410–411 K; *R*_f_ = 0.69 (hexane–ethyl acetate, 1:1 v/v)]. Single crystals suitable for X-ray diffraction were prepared by slow evaporation of a solution of the title compound in diisopropyl ether at room temperature.

### Refinement

All H atoms were positioned geometrically and refined using a riding model, with C—H = 0.95 Å for aryl and 0.98 Å for methyl H atoms. *U*_iso_(H) = 1.2*U*_eq_ (C) for aryl and 1.5*U*_eq_(C) for methyl H atoms.

### Crystal data

**Table:**

C_17_H_15_FO_2_S	*F*(000) = 632
*M**_r_* = 302.35	*D*_x_ = 1.386 Mg m^−^^3^
Orthorhombic, *P**n**a*2_1_	Mo *K*α radiation, λ = 0.71073 Å
Hall symbol: P 2c -2n	Cell parameters from 5305 reflections
*a* = 11.9486 (4) Å	θ = 2.8–31.0°
*b* = 18.8134 (9) Å	µ = 0.24 mm^−^^1^
*c* = 6.4435 (3) Å	*T* = 173 K
*V* = 1448.46 (11) Å^3^	Block, colourless
*Z* = 4	0.36 × 0.34 × 0.23 mm

### Data collection

**Table:**

Bruker SMART APEXII CCD diffractometer	2147 independent reflections
Radiation source: Rotating Anode	2094 reflections with *I* > 2σ(*I*)
Bruker HELIOS graded multilayer optics	*R*_int_ = 0.027
Detector resolution: 10.0 pixels mm^-1^	θ_max_ = 25.0°, θ_min_ = 2.0°
φ and ω scans	*h* = −13→14
Absorption correction: multi-scan (*SADABS*; Bruker, 2009)	*k* = −22→9
*T*_min_ = 0.920, *T*_max_ = 0.947	*l* = −5→7
6188 measured reflections	

### Refinement

**Table:**

Refinement on *F*^2^	Secondary atom site location: difference Fourier map
Least-squares matrix: full	Hydrogen site location: difference Fourier map
*R*[*F*^2^ > 2σ(*F*^2^)] = 0.029	H-atom parameters constrained
*wR*(*F*^2^) = 0.076	*w* = 1/[σ^2^(*F*_o_^2^) + (0.0501*P*)^2^ + 0.2112*P*] where *P* = (*F*_o_^2^ + 2*F*_c_^2^)/3
*S* = 1.05	(Δ/σ)_max_ < 0.001
2147 reflections	Δρ_max_ = 0.19 e Å^−^^3^
193 parameters	Δρ_min_ = −0.32 e Å^−^^3^
1 restraint	Absolute structure: Flack (1983), 745 Friedel pairs
Primary atom site location: structure-invariant direct methods	Flack parameter: 0.01 (8)

### Special details

**Table:**

Geometry. All esds (except the esd in the dihedral angle between two l.s. planes) are estimated using the full covariance matrix. The cell esds are taken into account individually in the estimation of esds in distances, angles and torsion angles; correlations between esds in cell parameters are only used when they are defined by crystal symmetry. An approximate (isotropic) treatment of cell esds is used for estimating esds involving l.s. planes.
Refinement. Refinement of F^2^ against ALL reflections. The weighted R-factor wR and goodness of fit S are based on F^2^, conventional R-factors R are based on F, with F set to zero for negative F^2^. The threshold expression of F^2^ > 2sigma(F^2^) is used only for calculating R-factors(gt) etc. and is not relevant to the choice of reflections for refinement. R-factors based on F^2^ are statistically about twice as large as those based on F, and R- factors based on ALL data will be even larger.

### Fractional atomic coordinates and isotropic or equivalent isotropic displacement parameters (Å^2^)

**Table:**

	*x*	*y*	*z*	*U*_iso_*/*U*_eq_	
S	0.58051 (3)	0.24745 (2)	0.81941 (12)	0.02603 (14)	
F	0.48280 (12)	0.04865 (7)	0.1409 (3)	0.0569 (4)	
O1	0.31672 (10)	0.36585 (6)	0.9109 (2)	0.0272 (3)	
O2	0.69067 (10)	0.28105 (7)	0.7715 (3)	0.0352 (4)	
C1	0.47298 (13)	0.31127 (8)	0.8012 (4)	0.0235 (4)	
C2	0.44816 (14)	0.36764 (9)	0.6520 (3)	0.0232 (4)	
C3	0.49438 (14)	0.39469 (9)	0.4673 (4)	0.0264 (4)	
C4	0.43695 (16)	0.45042 (9)	0.3733 (3)	0.0307 (5)	
H4	0.4664	0.4697	0.2485	0.037*	
C5	0.33771 (15)	0.47965 (10)	0.4533 (4)	0.0321 (5)	
C6	0.35091 (14)	0.39864 (9)	0.7305 (3)	0.0247 (4)	
C18	0.29371 (15)	0.45395 (9)	0.6362 (4)	0.0299 (4)	
H18	0.2274	0.4732	0.6948	0.036*	
C7	0.39288 (14)	0.31267 (9)	0.9495 (3)	0.0250 (4)	
C8	0.59776 (16)	0.36461 (11)	0.3682 (3)	0.0328 (5)	
H8A	0.5784	0.3213	0.2919	0.049*	
H8B	0.6529	0.3533	0.4759	0.049*	
H8C	0.6294	0.3996	0.2721	0.049*	
C9	0.27873 (19)	0.53802 (10)	0.3354 (5)	0.0480 (6)	
H9A	0.2007	0.5411	0.3816	0.072*	
H9B	0.2808	0.5275	0.1865	0.072*	
H9C	0.3164	0.5834	0.3616	0.072*	
C10	0.36932 (15)	0.26859 (10)	1.1344 (4)	0.0315 (4)	
H10A	0.2985	0.2432	1.1146	0.047*	
H10B	0.3638	0.2992	1.2571	0.047*	
H10C	0.4300	0.2342	1.1540	0.047*	
C11	0.54562 (14)	0.19223 (9)	0.6020 (3)	0.0247 (4)	
C12	0.44150 (15)	0.15884 (10)	0.5924 (4)	0.0316 (5)	
H12	0.3854	0.1694	0.6921	0.038*	
C13	0.42097 (15)	0.11030 (11)	0.4364 (4)	0.0367 (5)	
H13	0.3506	0.0870	0.4271	0.044*	
C14	0.50423 (17)	0.09628 (9)	0.2943 (4)	0.0367 (5)	
C15	0.60681 (16)	0.12901 (10)	0.2988 (4)	0.0340 (5)	
H15	0.6618	0.1191	0.1963	0.041*	
C16	0.62779 (14)	0.17706 (10)	0.4580 (4)	0.0299 (4)	
H16	0.6988	0.1995	0.4678	0.036*	

### Atomic displacement parameters (Å^2^)

**Table:**

	*U*^11^	*U*^22^	*U*^33^	*U*^12^	*U*^13^	*U*^23^
S	0.0224 (2)	0.0280 (2)	0.0277 (3)	0.00348 (15)	−0.0020 (2)	0.00368 (19)
F	0.0605 (8)	0.0523 (7)	0.0578 (11)	−0.0008 (6)	0.0001 (8)	−0.0262 (8)
O1	0.0251 (6)	0.0272 (6)	0.0294 (9)	0.0035 (4)	0.0020 (6)	−0.0003 (6)
O2	0.0207 (6)	0.0380 (7)	0.0469 (12)	−0.0016 (5)	−0.0048 (6)	0.0005 (7)
C1	0.0213 (7)	0.0236 (8)	0.0256 (11)	0.0000 (5)	−0.0015 (8)	0.0000 (8)
C2	0.0232 (7)	0.0227 (8)	0.0237 (10)	−0.0013 (6)	−0.0046 (8)	0.0008 (8)
C3	0.0277 (8)	0.0250 (9)	0.0264 (11)	−0.0062 (7)	−0.0046 (8)	0.0008 (8)
C4	0.0345 (9)	0.0278 (9)	0.0297 (13)	−0.0086 (7)	−0.0045 (9)	0.0064 (8)
C5	0.0313 (9)	0.0240 (9)	0.0411 (15)	−0.0026 (7)	−0.0115 (9)	0.0061 (9)
C6	0.0249 (8)	0.0232 (8)	0.0259 (11)	−0.0031 (6)	−0.0025 (8)	−0.0007 (7)
C18	0.0261 (8)	0.0224 (8)	0.0412 (13)	0.0013 (7)	−0.0048 (9)	−0.0016 (9)
C7	0.0236 (7)	0.0250 (8)	0.0263 (11)	−0.0003 (6)	−0.0036 (8)	0.0018 (8)
C8	0.0325 (9)	0.0369 (10)	0.0290 (14)	−0.0046 (7)	0.0037 (9)	0.0051 (9)
C9	0.0476 (12)	0.0365 (11)	0.0599 (19)	0.0043 (8)	−0.0101 (13)	0.0178 (12)
C10	0.0288 (9)	0.0373 (9)	0.0282 (12)	−0.0003 (8)	0.0027 (9)	0.0049 (10)
C11	0.0231 (8)	0.0227 (8)	0.0284 (12)	0.0047 (7)	0.0007 (8)	0.0058 (8)
C12	0.0261 (9)	0.0290 (9)	0.0398 (14)	−0.0007 (7)	0.0076 (9)	0.0029 (9)
C13	0.0320 (10)	0.0331 (10)	0.0449 (16)	−0.0055 (7)	0.0023 (10)	−0.0033 (10)
C14	0.0407 (10)	0.0287 (9)	0.0408 (15)	0.0047 (7)	0.0001 (10)	−0.0041 (10)
C15	0.0337 (9)	0.0356 (10)	0.0328 (13)	0.0087 (7)	0.0069 (11)	0.0006 (10)
C16	0.0227 (8)	0.0311 (9)	0.0360 (13)	0.0038 (7)	0.0035 (9)	0.0048 (9)

### Geometric parameters (Å, °)

**Table:**

S—O2	1.4925 (13)	C8—H8A	0.9800
S—C1	1.7625 (16)	C8—H8B	0.9800
S—C11	1.793 (2)	C8—H8C	0.9800
F—C14	1.358 (3)	C9—H9A	0.9800
O1—C7	1.375 (2)	C9—H9B	0.9800
O1—C6	1.378 (2)	C9—H9C	0.9800
C1—C7	1.353 (3)	C10—H10A	0.9800
C1—C2	1.462 (3)	C10—H10B	0.9800
C2—C6	1.395 (3)	C10—H10C	0.9800
C2—C3	1.407 (3)	C11—C16	1.380 (3)
C3—C4	1.392 (3)	C11—C12	1.395 (2)
C3—C8	1.501 (3)	C12—C13	1.380 (3)
C4—C5	1.405 (3)	C12—H12	0.9500
C4—H4	0.9500	C13—C14	1.377 (3)
C5—C18	1.378 (3)	C13—H13	0.9500
C5—C9	1.510 (3)	C14—C15	1.372 (3)
C6—C18	1.385 (3)	C15—C16	1.390 (3)
C18—H18	0.9500	C15—H15	0.9500
C7—C10	1.478 (3)	C16—H16	0.9500
			
O2—S—C1	109.91 (8)	H8A—C8—H8C	109.5
O2—S—C11	106.78 (9)	H8B—C8—H8C	109.5
C1—S—C11	99.97 (9)	C5—C9—H9A	109.5
C7—O1—C6	106.39 (14)	C5—C9—H9B	109.5
C7—C1—C2	107.87 (15)	H9A—C9—H9B	109.5
C7—C1—S	118.82 (15)	C5—C9—H9C	109.5
C2—C1—S	133.31 (15)	H9A—C9—H9C	109.5
C6—C2—C3	118.82 (16)	H9B—C9—H9C	109.5
C6—C2—C1	103.55 (17)	C7—C10—H10A	109.5
C3—C2—C1	137.63 (17)	C7—C10—H10B	109.5
C4—C3—C2	116.55 (18)	H10A—C10—H10B	109.5
C4—C3—C8	120.3 (2)	C7—C10—H10C	109.5
C2—C3—C8	123.12 (17)	H10A—C10—H10C	109.5
C3—C4—C5	123.5 (2)	H10B—C10—H10C	109.5
C3—C4—H4	118.3	C16—C11—C12	120.76 (19)
C5—C4—H4	118.3	C16—C11—S	118.65 (14)
C18—C5—C4	119.89 (18)	C12—C11—S	120.18 (16)
C18—C5—C9	120.50 (19)	C13—C12—C11	119.26 (19)
C4—C5—C9	119.6 (2)	C13—C12—H12	120.4
O1—C6—C18	124.05 (17)	C11—C12—H12	120.4
O1—C6—C2	111.43 (15)	C14—C13—C12	118.84 (17)
C18—C6—C2	124.49 (19)	C14—C13—H13	120.6
C5—C18—C6	116.76 (17)	C12—C13—H13	120.6
C5—C18—H18	121.6	F—C14—C15	118.7 (2)
C6—C18—H18	121.6	F—C14—C13	118.28 (18)
C1—C7—O1	110.76 (17)	C15—C14—C13	123.0 (2)
C1—C7—C10	133.87 (17)	C14—C15—C16	118.0 (2)
O1—C7—C10	115.33 (16)	C14—C15—H15	121.0
C3—C8—H8A	109.5	C16—C15—H15	121.0
C3—C8—H8B	109.5	C11—C16—C15	120.13 (17)
H8A—C8—H8B	109.5	C11—C16—H16	119.9
C3—C8—H8C	109.5	C15—C16—H16	119.9
			
O2—S—C1—C7	−136.51 (15)	C9—C5—C18—C6	177.70 (19)
C11—S—C1—C7	111.44 (17)	O1—C6—C18—C5	−178.39 (17)
O2—S—C1—C2	43.3 (2)	C2—C6—C18—C5	−0.5 (3)
C11—S—C1—C2	−68.77 (19)	C2—C1—C7—O1	−0.2 (2)
C7—C1—C2—C6	0.1 (2)	S—C1—C7—O1	179.66 (12)
S—C1—C2—C6	−179.73 (15)	C2—C1—C7—C10	177.4 (2)
C7—C1—C2—C3	−179.6 (2)	S—C1—C7—C10	−2.8 (3)
S—C1—C2—C3	0.6 (4)	C6—O1—C7—C1	0.2 (2)
C6—C2—C3—C4	−1.3 (3)	C6—O1—C7—C10	−177.86 (16)
C1—C2—C3—C4	178.3 (2)	O2—S—C11—C16	13.49 (17)
C6—C2—C3—C8	−179.13 (17)	C1—S—C11—C16	127.95 (15)
C1—C2—C3—C8	0.5 (4)	O2—S—C11—C12	−173.86 (14)
C2—C3—C4—C5	−0.1 (3)	C1—S—C11—C12	−59.40 (17)
C8—C3—C4—C5	177.82 (18)	C16—C11—C12—C13	−0.2 (3)
C3—C4—C5—C18	1.2 (3)	S—C11—C12—C13	−172.66 (16)
C3—C4—C5—C9	−177.43 (19)	C11—C12—C13—C14	0.0 (3)
C7—O1—C6—C18	178.01 (18)	C12—C13—C14—F	−179.69 (19)
C7—O1—C6—C2	−0.16 (19)	C12—C13—C14—C15	−0.8 (4)
C3—C2—C6—O1	179.81 (15)	F—C14—C15—C16	−179.36 (19)
C1—C2—C6—O1	0.05 (19)	C13—C14—C15—C16	1.7 (3)
C3—C2—C6—C18	1.7 (3)	C12—C11—C16—C15	1.2 (3)
C1—C2—C6—C18	−178.11 (18)	S—C11—C16—C15	173.77 (15)
C4—C5—C18—C6	−1.0 (3)	C14—C15—C16—C11	−1.9 (3)

### Hydrogen-bond geometry (Å, °)

**Table:**

Cg is the centroid of the C11–C16 ring.

**Table:**

*D*—H···*A*	*D*—H	H···*A*	*D*···*A*	*D*—H···*A*
C10—H10A···O2^i^	0.98	2.60	3.301 (3)	129
C10—H10C···Cg^ii^	0.98	2.78	3.604 (3)	142

Symmetry codes: (i) *x*−1/2, −*y*+1/2, *z*; (ii) *x*, *y*, *z*+1.
